# Robotic Arm Trajectory Planning in Dynamic Environments Based on Self-Optimizing Replay Mechanism

**DOI:** 10.3390/s25154681

**Published:** 2025-07-29

**Authors:** Pengyao Xu, Chong Di, Jiandong Lv, Peng Zhao, Chao Chen, Ruotong Wang

**Affiliations:** 1 Shandong Artificial Intelligence Institute, Qilu University of Technology (Shandong Academy of Sciences), Jinan 250014, China; xupy@sdas.org (P.X.); dich@sdas.org (C.D.); zhaopeng2@sd.chinamobile.com (P.Z.); 2 College of Computer Science and Engineering, Shandong University of Science and Technology, Qingdao 266590, China

**Keywords:** experience replay mechanism, reward function design, dynamic environment, robotic manipulation

## Abstract

In complex dynamic environments, robotic arms face multiple challenges such as real-time environmental changes, high-dimensional state spaces, and strong uncertainties. Trajectory planning tasks based on deep reinforcement learning (DRL) suffer from difficulties in acquiring human expert strategies, low experience utilization (leading to slow convergence), and unreasonable reward function design. To address these issues, this paper designs a neural network-based expert-guided triple experience replay mechanism (NETM) and proposes an improved reward function adapted to dynamic environments. This replay mechanism integrates imitation learning’s fast data fitting with DRL’s self-optimization to expand limited expert demonstrations and algorithm-generated successes into optimized expert experiences. Experimental results show the expanded expert experience accelerates convergence: in dynamic scenarios, NETM boosts accuracy by over 30% and safe rate by 2.28% compared to baseline algorithms.

## 1. Introduction

In recent years, robots have become core tools in high-end equipment and intelligent manufacturing, and their research, development, manufacturing, and application have become important indicators to measure a country’s scientific and technological innovation capabilities and manufacturing level [1,2]. The application of DRL in the field of robotic arm trajectory planning is gradually becoming a new research hotspot [3]. This method imitates the human process of learning to use arms, relying not on traditional teaching or complex algorithms but on continuous interaction and trial-and-error with the environment to gradually learn operational skills. DRL-based robotic arm control methods have demonstrated good generalization ability, robustness, and adaptability. Despite the challenges posed by the robotic arm’s high-dimensional state-action space, DRL integrating deep learning’s perception with reinforcement learning’s decision-making has proven effective for robotic arm intelligence [4].

## 2. Related Work

Gu et al. [5] optimized the output of the traditional DQN network to address the limitations of deep Q-learning in handling continuous action spaces, proposing a Q-learning algorithm suitable for continuous action spaces: Normalized Advantage Functions (NAFs), which expand the application range of DQN to robotic arm control tasks. Mahmood et al. [6] used four algorithms, including Deep Deterministic Policy Gradient, Trust Region Policy Optimization, Soft Q-Learning, and Proximal Policy Optimization, to achieve the motion control of a 6-DoF robotic arm. In the experiment, these algorithms were used to control two and six joints of the robotic arm, respectively. The results show that the fewer the number of controlled joints of the robotic arm, the better the effect of the DRL algorithm; conversely, as the number of joints increases, the difficulty of learning effective strategies also increases. Lim et al. [7] proposed a method using sparse Gaussian processes to predict unknown reward functions, which trains the model using trajectory-reward pairs generated by DRL with different reward functions, capable of accurately inferring reward functions from very few human expert demonstration data. Qi et al. [8] proposed an improved Deep Deterministic Policy Gradient algorithm. Firstly, aiming at the shortcomings of the traditional DDPG reward function, they designed a composite reward function. Secondly, to address the low sampling efficiency of the robotic arm, they added a batch of teaching data to the experience replay pool to improve learning efficiency. They applied the improved DDPG algorithm to the robotic arm grasping task, and the grasping accuracy was significantly improved. Li et al. [9] proposed a DRL algorithm with automatic entropy adjustment based on the maximum entropy framework and Tsallis entropy, enabling the robotic arm to cope with diverse tasks. Moreover, they also proposed an improved path planning method combining traditional path planning methods and DRL, which improves planning performance. Ma et al. [10] proposed a new framework using a small amount of demonstration data to guide exploration to solve the problem of slow convergence caused by random and unnecessary exploration. Among them, they used a neural network to model the demonstration data as a function between directional preference and distance to the target state. Ying et al. [11] proposed a dual experience replay pool structure strategy, which improves the early training effect by using successful experiences and unbiased data sampling. Meanwhile, they also developed a fuzzy feedback reward mechanism to establish a direct mapping from state variables to rewards. Tang W et al. [12] studied the trajectory planning of dual-arm robots based on deep reinforcement learning in complex environments. To address the issue of sparse rewards that robots obtain from the environment, they employed the Proximal Policy Optimization (PPO) algorithm with a continuous reward function to train a neural network for controlling the robotic arm. Inspired by the concept of the artificial potential field, they proposed new reward and punishment functions, including a reward guidance function, collision detection, an obstacle avoidance function, and a time function. Compared with the DDPG algorithm, the PPO algorithm reduced the number of training convergence steps by approximately 4 million, verifying the advantages of the algorithm. J. Heaton et al. [13] applied DRL to the motion planning of robotic arms. In their work, they used the SAC network to train the model, guiding the robotic arm to reach different positions and preventing collisions between its end-effector or the carried objects and the “game tower”. This demonstrates a simple and effective method for training agents to achieve goals, and the method can be extended to similar yet different environments. Wu et al. [14] pointed out that robot decision-making in dynamic environments needs to balance real-time performance and robustness, and the discrepancy between the simulation environment and real scenarios is the core bottleneck restricting the implementation of algorithms. Wang et al. [15] proposed a method for designing action trigger conditions in multi-agent reinforcement learning based on genetic programming, emphasizing the adaptability of dynamic strategy adjustment to complex tasks, which provides cross-domain ideas for optimizing the dynamic sampling strategy in the NETM mechanism of this paper. Fang et al. [16] noted in their review that imitation learning in robotic arm operations faces the contradiction between the quality of expert demonstration data and scene generalization, while this paper adopts the method of expanding expert experience through GAIL.

During the training of off-policy [17,18] algorithms, especially in the early training stage, policy optimization is very slow, and researchers usually use the Prioritized Experience Replay (PER) mechanism to accelerate training. This method intelligently selects more valuable samples for focused training, improving the utilization efficiency of experience data, but its effect highly depends on high-quality data. When the data quality is low, even sampling by priority cannot effectively improve the learning efficiency and performance of the algorithm. In the early training stage of trajectory planning tasks, the robotic arm mainly interacts with the environment through random exploration, leading to the accumulation of a large amount of low-quality data in the experience pool, especially in dynamic, unstructured environments. Osa T et al. [19] proposed an expert-guided policy learning method by adding additional expert experience data to the experience replay pool to accelerate the learning process. However, there are problems such as high time and cost for high-quality human expert demonstrations, non-optimal trajectories in expert operations and decisions, resulting in limited knowledge of the robotic arm that cannot learn better strategies, and slight changes in the actual environment affecting the effectiveness of previously learned strategies, necessitating re-adaptation to new environment expert strategies.

To select high-value experiences for learning [20], PER needs to calculate the priority sampling weights of experience data, significantly increasing the time complexity of the algorithm. At the same time, PER also introduces new hyperparameters [21], and finding appropriate hyperparameter settings requires a lot of trials and adjustments, which is tedious and time-consuming. Therefore, an experience replay mechanism with a small amount of expert experience guidance, low time complexity, and no introduction of hyperparameters is particularly important.

Finally, in the robotic arm obstacle avoidance trajectory planning task, a more general reward function can significantly improve the efficiency of the robotic arm in autonomously generating obstacle-avoiding trajectories [22]. Currently, the design of reward functions for obstacle avoidance usually adopts the idea of the artificial potential field method, which has a weak perception of dynamic obstacles and poor performance in the face of dynamic targets, exacerbating the difficulty of algorithm learning. Therefore, it is necessary to design a general reward function suitable for the robotic arm dynamic obstacle avoidance trajectory planning task [23].

Although DRL has made certain progress in robotic arm control, it still has problems such as poor algorithm training efficiency and difficult reward function design, with great development potential.

Aiming at the above problems, this paper first proposes a NETM. The main innovations of this method are as follows:

- Expanding a small amount of human expert demonstrations and successful experiences generated by the algorithm into a large number of expert experiences, enabling the robotic arm to obtain a large amount of high-quality experience data throughout the entire training phase.
- Designing a dynamic sampling strategy to divide the experiences generated by the algorithm into successful experiences and failed experiences, then using a neural network to dynamically extract experiences from successful experiences, failed experiences, and expert experiences at different ratios in different training stages, integrating expert experiences into the algorithm while improving the utilization rate of experience data. NETM does not introduce additional hyperparameters and has relatively low time complexity.
- For moving targets in trajectory planning, adding velocity constraints between the end-effector and the moving target to the gravitational potential field of the artificial potential field method, canceling the action range of the obstacle repulsive potential field for dynamic obstacles in the environment, and proposing a generalized safety reward (GSR) suitable for dynamic environments based on this.

The first chapter of this paper introduces the research background, followed by an introduction to related work. The third part presents the model methodology, and the fourth part describes the experimental setup and results. Finally, the research work is summarized.

## 3. Method Introduction

**Figure 1:**
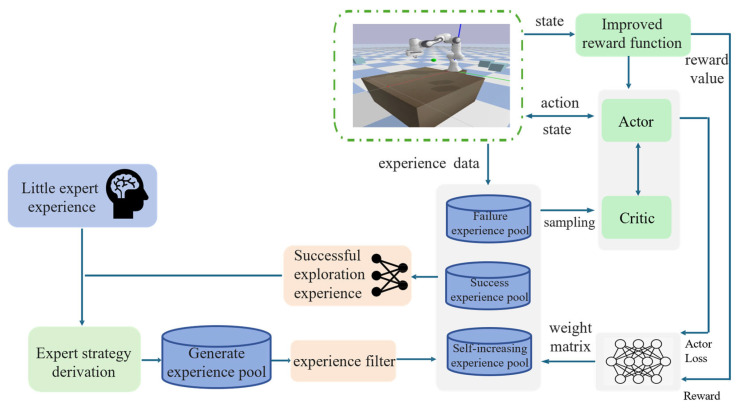
Dynamic obstacle avoidance trajectory planning framework.

This paper will detail the proposed DRL-based dynamic obstacle avoidance trajectory planning framework for robotic arms, as shown in Figure 1. It consists of two parts: the NETM and the reward function suitable for dynamic environments.

### 3.1. Neural Network-Based Expert-Guided Experience Sampling Strategy

The NETM includes three parts: expert demonstration, expert policy derivation, and a neural network-based experience sampling strategy. In the expert demonstration part, a small amount of demonstration data can be obtained through human expert demonstration, traditional inverse kinematics-based trajectory planning, or other human expert-based technologies; in the expert policy derivation part, Generative Adversarial Imitation Learning (GAIL) is used to augment the expert demonstration part and successful experiences generated by the algorithm, and a self-increasing expert experience replay pool is set up to store the generated expert experiences in the self-increasing expert experience replay pool; in the neural network-based experience sampling strategy part, the original experience pool is divided into a successful experience replay pool and a failed experience replay pool, and a neural network is used to autonomously learn a weight matrix attached to the self-increasing expert experience replay pool, successful experience replay pool, and failed experience replay pool, dynamically sampling different types of experience data at different ratios according to different stages of DRL algorithm training to improve the utilization rate of experience data.

#### 3.1.1. Expert Policy Derivation

Before deriving the expert policy, the expert trajectory sample set in the expert demonstration part is first defined as

(1) $$RM_{e}=\left\{\Psi_{1},\Psi_{2},\cdots ,\Psi_{i},\cdots ,\Psi_{N}\right\}$$

where $RM_{e}$ is the expert trajectory sample set, $\Psi_{i}$ is the i-th expert trajectory sample, N is the number of expert trajectory samples, and $\Psi_{i}$ can be further transformed into a set of state-action pairs $\left(s_{t},a_{t}\right)$.

For expert policy derivation, we establish a self-increasing expert pool $M_{e}$ and use GAIL (Generative Adversarial Network (GAN) [24] and Imitation Learning (IL) [25]) to augment expert trajectories and algorithm-generated successes. The augmented data are stored in $M_{e}$, where GAN’s adversarial training generates expert-like behavior [26].

In GAIL, there are two main structures: generator G and discriminator $D(s_{t},a_{t}|\theta^{D})$. The discriminator D is composed of a three-layer fully connected network, which takes the state-action pairs generated by the generator G as negative samples and the state-action pairs generated by expert trajectory data and successful data from the algorithm as positive samples, outputting the similarity between positive and negative samples, denoted as $D_{t}$, $D_{t}$, with a value range of $\left[0,\;1\right]$. The closer the action generated by the generator is to the expert action, the closer the value of $D_{t}$ is to 1. To better distinguish between the behavior generated by the generator and expert behavior, the optimization goal of the discriminator D is to minimize

(2) $$\nabla_{\theta }^{D}D{|}_{s_{i},a_{i}}={\hat{E}}_{T_{G}}\left[\nabla_{\theta }^{D}log\left(D\left(s_{i},a_{i}\right)\right)\right]+{\hat{E}}_{RM_{e}}\left[\nabla_{\theta }^{D}log\left(1-D\left(s_{i},a_{i}\right)\right)\right]$$

where $T_{G}$ is the trajectory sample set obtained from the generator.

The generator G can be any conventional reinforcement learning algorithm (such as DDPG, SAC), taking the current environmental state $s_{t}$ as the input, the output $D_{t}$ of the discriminator as the reward function, and outputting actions $a_{t}$ that are increasingly similar to experts.

**Figure 2:**
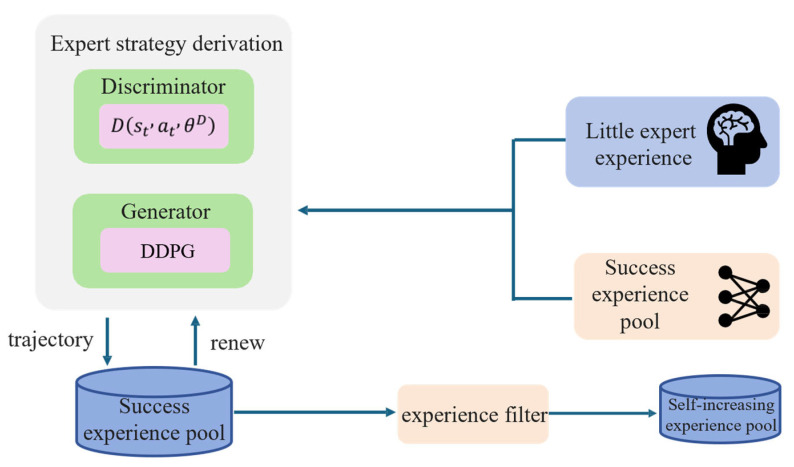
Self-increasing expert experience replay pool.

In GAIL, the discriminator and generator are trained alternately, with the parameters of the generator fixed during discriminator training and vice versa. Through this adversarial process, the generator continuously learns and improves to produce behavior increasingly close to the expert level. Therefore, even with only a small amount of expert experience initially, GAIL can generate a large amount of high-quality experience data through internal learning processes. As shown in Figure 2, before GAIL training, expert experiences obtained from expert teaching are used as positive samples for the discriminator. Then, in each round of GAIL training, the generator attempts to imitate expert behavior and generates an entire trajectory stored in the generated experience replay pool.

However, these generated trajectories are not always high-quality, successful trajectories, and their guiding effect on the algorithm is limited. Therefore, this paper sets up an experience filter to screen successful (where the end-effector can reach near the target point and maintain around the target) trajectories into the self-increasing expert experience replay pool $M_{e}$. The principle of the experience filter is the state-action pairs generated by the generator are passed to the robotic arm in chronological order, and the environment’s feedback is used to determine whether the task is completed. The self-increasing expert experience pool $M_{e}$ uses a first-in-first-out update method. When the number of experiences in the experience replay pool exceeds the set maximum capacity of the experience pool, newly added data gradually replaces the earliest data. As training progresses, the generator’s ability will continuously improve during the adversarial process, learning behavior patterns closer to expert strategies, thus gradually improving the quality of generated data.

It is worth noting that some expert trajectories are not optimal, which may cause the robotic arm to learn suboptimal strategies. Therefore, this paper also inputs the successful experiences generated during DRL algorithm training into GAIL for augmentation to ensure the quality of experiences in the self-increasing expert experience replay pool. In the early stage of DRL training, due to the extremely fast training speed of GAIL, the self-increasing expert experience replay pool will quickly be filled with a large number of successful trajectories. Regardless of the quality of these successful trajectories, they will have a significant guiding effect on the robotic arm’s training. As training progresses, DRL gradually converges, generating more and more successful experiences, which will gradually dominate the positive samples of the discriminator, and the quality of these experiences is also continuously optimized, which will gradually weaken the influence of some low-quality expert experiences, thereby improving the data quality in the self-increasing expert experience replay pool and providing stronger guidance for the robotic arm.

#### 3.1.2. Neural Network-Based Experience Sampling Strategy

To introduce expert experience while improving experience utilization, this section proposes a neural network-based experience sampling strategy [27]. This strategy first divides the original experience replay pool into two parts: a successful experience replay pool $M_{success}$ and a failed experience replay pool $M_{fail}$. Experiences corresponding to the robotic arm successfully completing tasks are stored in the successful experience replay pool, and failed experiences are stored in the failed experience replay pool. Due to the delayed reward of reinforcement learning, previous experience data also need to be stored in the relevant experience replay pool. Therefore, this paper adds a cache pool with a capacity of the maximum time step. When an episode ends, all relevant experiences during this period (i.e., experiences in the cache pool) are transferred to the corresponding experience replay pool according to the result. For example, if the robotic arm successfully completes the task, all experiences during this period will be transferred from the cache pool to the successful experience replay pool; conversely, if the task is not completed, these experiences will be transferred to the failed experience replay pool.

During DRL algorithm training, the value of different types of experience data is different, so NETM extracts experiences from the three experience replay pools at different ratios for learning. Moreover, the value of the three experiences dynamically changes with different training stages, so the sampling ratio should also change with the training stage. If the sampling ratio is given before the algorithm starts training, it will affect the algorithm’s convergence speed and final performance. To maximize the utilization of experience data, this paper learns a weight coefficient matrix $\alpha =\left[\alpha_{z},\;\alpha_{success},\;\alpha_{fail}\right]$ through a neural network and attaches it to the self-increasing expert experience replay pool, successful experience replay pool, and failed experience replay pool, enabling the algorithm to dynamically control the proportion of extracted experiences according to the training progress:

(3) $$\left\{\begin{array}{c} N=\alpha_{z}\times N+\alpha_{success}\times N+\alpha_{fail}\times N\\ \alpha_{z}+\alpha_{success}+\alpha_{fail}=1 \end{array}\right.$$

**Figure 3:**
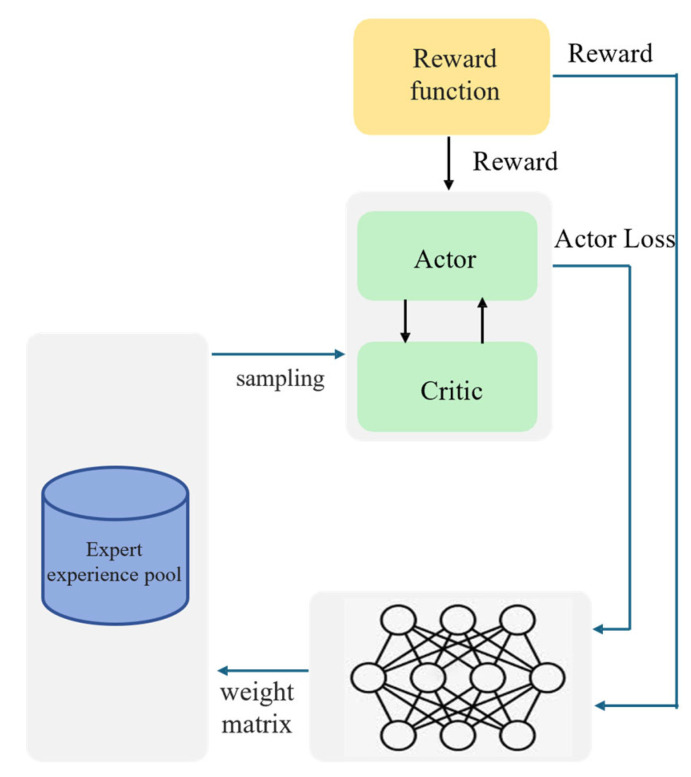
Experience sampling strategy based on neural network.

The designed neural network for learning the weight coefficient matrix α is shown in Figure 3. To dynamically adjust the sampling ratio, NETM uses a neural network to learn the weight coefficient matrix α using existing knowledge, where the neural network parameters are ω. In the algorithms DDPG and SAC used in this paper, the algorithm’s reward value and the loss value of the policy network are important indicators reflecting the algorithm’s performance and the optimization degree of the policy network, as well as valuable existing knowledge. Therefore, this paper uses the algorithm’s reward value and the loss value of the policy network as the input of the neural network. To maximize the reward, the loss of the neural network is defined as the opposite of the reward value:

(4) $$J\left(\begin{array}{c} \omega \end{array}\right)=-R$$

### 3.2. Reward Function Based on Improved Artificial Potential Field Method in Dynamic Environment

In DRL algorithms, designing an efficient reward function is crucial. Currently, reward functions for obstacle avoidance usually adopt the idea of the artificial potential field method, that is, attaching a potential field to the environment, which includes a gravitational potential field generated by the target position to guide the robotic arm to move toward the target position and a repulsive potential field generated by obstacles to guide the robotic arm to avoid obstacles [28]. Generally, the original reward function (OSR) [29], defined based on this idea, usually takes the following form:

(5) $$R_{f}=-R_{T}-R_{O}$$

where $R_{T}$ is the gravitational potential field reward function and $R_{O}$ is the repulsive potential field reward function. $R_{T}$ is provided by the distance between the robotic arm’s end-effector and the target position: the greater the distance, the greater the gravitational force, and vice versa.

When the end-effector reaches the target position, the gravitational force is 0. The gravitational potential field reward function is defined as

(6) $$R_{T}=c_{1}d_{T}^{2}$$

where $c_{1}$ is the gravitational potential field coefficient and $d_{T}$ is the distance between the end-effector and the target position.

$R_{O}$ is provided by the minimum distance between the robotic arm and obstacles: the greater the distance, the smaller the repulsive force, and vice versa. Existing work usually sets an obstacle influence range for the penalty term, called the local repulsive potential field reward function, generally defined as

(7) $$R_{o}=\left\{\begin{array}{c} c_{2}\left(\frac{\varphi }{d_{O}}-1\right),d_{O}\leq \varphi \\ 0,d_{O}>\varphi \end{array}\right.$$

where $c_{2}$ is the repulsive potential field coefficient, $d_{O}$ is the closest distance between the robotic arm and the obstacle, and φ is the obstacle influence range radius. $R_{o}$ only takes effect when the distance between the robotic arm and the obstacle is less than φ.

Although the effect of OSR has been verified in simple scenarios (static targets and static obstacles), it often performs poorly in dynamic environments. Therefore, this paper improves this reward function for dynamic environments. First, for moving targets in the environment, in addition to distance information, their velocity information relative to the end-effector is added to the gravitational potential field reward function to keep the movement trend of the end-effector consistent with that of the target:

(8) $$R_{TV}=c_{1}d_{T}^{2}+c_{3}{\left|v_{e}-v_{t}\right|}^{2}$$

where $c_{3}$ is the velocity influence factor, and $v_{e}$ and $v_{t}$ are the velocities of the end-effector and the target, respectively.

Second, in the local repulsive potential field reward function, the penalty term only affects the robotic arm’s training when $d_{O}$ is less than φ [30]. This obstacle avoidance penalty term cannot timely reflect the position change of dynamic obstacles when facing dynamic obstacles. Therefore, this paper designs a global repulsive potential field reward function that can sense dynamic obstacles in the environment:

(9) $$R_{OG}=c_{2}{\left(\frac{1}{1+d_{O}}\right)}^{n}$$

where the exponent n is also adjusted according to specific tasks. For the entire training process, $R_{OG}$ provides the robotic arm with global obstacle perception capability.

In addition, when the end-effector reaches the target position, the distance $d_{T}$ between the end-effector and the target position needs to be maintained within a certain range. At this time, the change in $d_{T}$ will be very small, which will cause the robotic arm to be insufficiently sensitive to the subtle position changes of the end-effector. Therefore, to learn these subtle action changes and make the end-effector continuously move toward the target position, this section adds an additional reward to the reward function:

(10) $$R_{1}=\left\{\begin{array}{c} \tau ,\;d_{T}^{n}<d_{T}^{n-1}\\ 0,d_{T}^{n}=d_{T}^{n-1}\\ -\tau ,d_{T}^{n}>d_{T}^{n-1} \end{array}\right.$$

where τ is a constant greater than zero, and $d_{T}^{n}$ is the distance between the end-effector and the target at the n-th time step.

In addition, for time steps where the end-effector position is maintained near the target, a sparse reward is additionally defined for it:

(11) $$R_{2}=\left\{\begin{array}{c} \delta ,d_{T}^{n}\leq \varepsilon \\ 0,d_{T}^{n}>\varepsilon \end{array}\right.$$

where δ is a constant greater than zero, and ε is the maximum allowable position error.

In summary, the GSR is defined as

(12) $$R_{t}=R_{TV}+R_{OG}+R_{1}+R_{2}$$

### 3.3. State Space and Action Space Design

The state space of the robotic arm refers to the sum of the environmental state and the robotic arm’s own state, which provides all necessary information for the robotic arm to make decisions and execute actions. In this paper, a 7-DoF robotic arm is used for experiments. If the joint angles and velocities of the robotic arm are directly used as observations, the learning efficiency of the robotic arm will be very low, and it may even fail to find the optimal trajectory [31]. Therefore, this paper sets the position $p_{e}$ and velocity $\dot{p_{e}}$ of the end-effector as observations, and then calculates the joint angles through Inverse Kinematics (IK) control. In addition, for the environmental state, this paper uses the relative positions ${\bar{p}}_{o}/{\bar{p}}_{t}$ and velocities ${\dot{\bar{p}}}_{o}/{\dot{\bar{p}}}_{t}$ of obstacles and targets relative to the end-effector to replace their absolute positions and velocities. Therefore, the state space S is defined as

(13) $$S=\left\{p_{e},{\dot{p}}_{e},{\bar{p}}_{t},{\dot{\bar{p}}}_{t},{\bar{p}}_{o},{\dot{\bar{p}}}_{o}\right\}$$

Since this paper sets the position of the end-effector as the observation, the dimension of the action space A can be reduced from seven to three:

(14) $$A=\left\{\Delta x,\Delta y,\Delta z\right\}$$

where Δx,Δy,Δz are the movement amounts of the robotic arm’s end-effector in the *X*, *Y*, and *Z* axis directions. To avoid sudden movement of the robotic arm due to excessive action output in a single time step, the value range of Δx,Δy,Δz is limited to −0.1 to 0.1.

## 4. Experimental Setup and Result Analysis

### 4.1. Parameter and Task Settings

**Figure 4:**
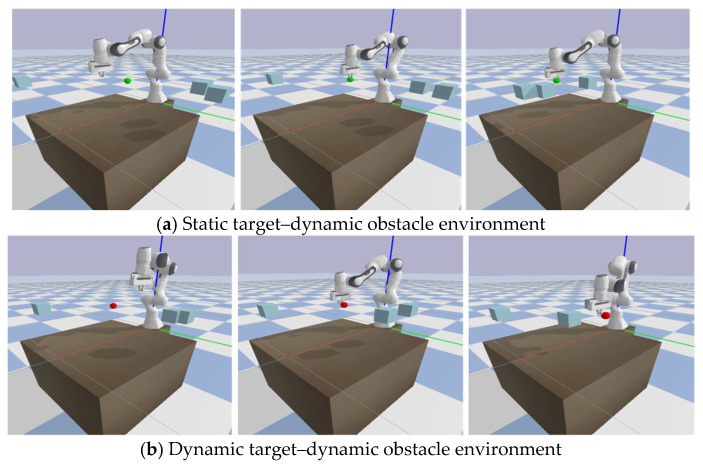
Two simulation environments.

This paper builds a simulation experiment environment for the Franka Emika Panda 7-DoF robotic arm(Franka Emika GmbH, Munich, Bavaria, Germany) based on the Pybullet platform(Google LLC, Mountain View, CA, USA) [32,33]. As shown in Figure 4, to evaluate the proposed method, two evaluation scenarios are set up: (1) static target–dynamic obstacle and (2) dynamic target–dynamic obstacle. In Scenario 1, the task goal is for the robotic arm to reach and maintain near the static target (green sphere) for as long as possible. In Scenario 2, the task goal is for the robotic arm to reach and follow the dynamic target (red sphere) for as long as possible. In both scenarios, there are three cube obstacles moving at a constant speed, and the robotic arm needs to avoid these obstacles. In addition, to ensure the model’s learning ability under uncertain conditions, the initial positions of the target and the three obstacles are uniformly randomly sampled within a certain range, and the initial position and movement range of the target are restricted to the reachable workspace of the robotic arm.

To achieve full-body obstacle avoidance for the robotic arm and obtain collision distances, this paper uses the Relation Subgraph Neural Network (RSGNet), where the robotic arm is set as a whole, and the three cube obstacles and the table in the scene are set as obstacles. RSGNet will detect the minimum distance between the robotic arm and obstacles in real-time. The main hyperparameter settings during the experiment are presented in Table 1. It should be noted that the Tesla A100 GPU utilized in this experiment is manufactured by NVIDIA Corporation, with its headquarters located in Santa Clara, California, United States.

### 4.2. Evaluation Indicators

To evaluate the effectiveness of the proposed experience replay mechanism and improved reward function, this paper uses two evaluation indicators: safe rate [34] and accuracy, which are defined as follows:

(15) $$Safe\;rate=\;\frac{No\;collision\;time\;steps}{Total\;time\;steps}\;\;\;$$

The safe rate is the proportion of time steps without collision in an episode to the total number of time steps.

(16) $$Accuracy=\frac{Success\;time\;steps}{Total\;time\;steps}$$

Accuracy refers to the proportion of successful time steps where the distance between the robotic arm’s end-effector position and the target position is kept within 0.05 m to the total number of time steps. Since the total number of time steps must include the time steps from the initial position to the target position of the robotic arm, the accuracy cannot reach 100%.

### 4.3. Experimental Result Analysis and Discussion

**Figure 5:**
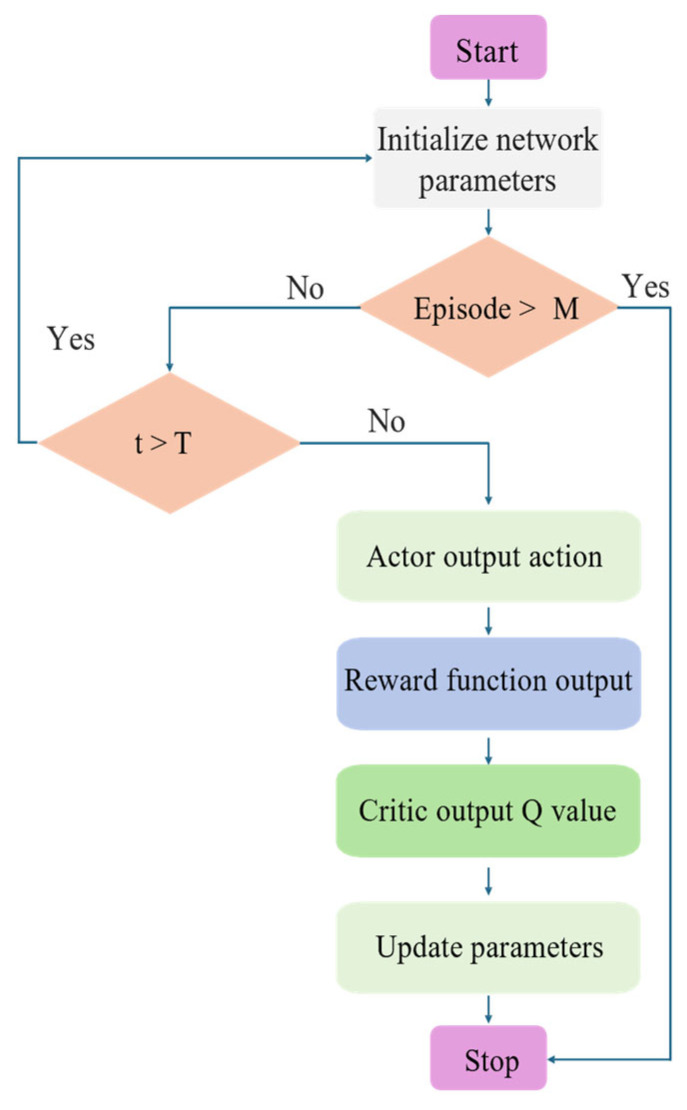
Schematic diagram of experimental training process.

In the experiment of this paper, each episode includes 100 time steps. After reaching 100 time steps, regardless of the result, the next episode of training starts immediately. Collision detection is performed at each time step. If a collision occurs, the number of collision time steps increases by 1. At the end of the episode, the safe rate is calculated based on the number of collision time steps. In this paper, the threshold ε of the collision detection model RSGNet is set to 0. If the distance between the end-effector position and the target position is less than 0.05 m at a certain time step, the number of successful time steps increases by 1. At the end of the episode, the accuracy is calculated based on the number of successful time steps. The training process of the experiment is shown in Figure 5.

#### 4.3.1. Comparison of Experience Replay Mechanism Experimental Effects

**Figure 6:**
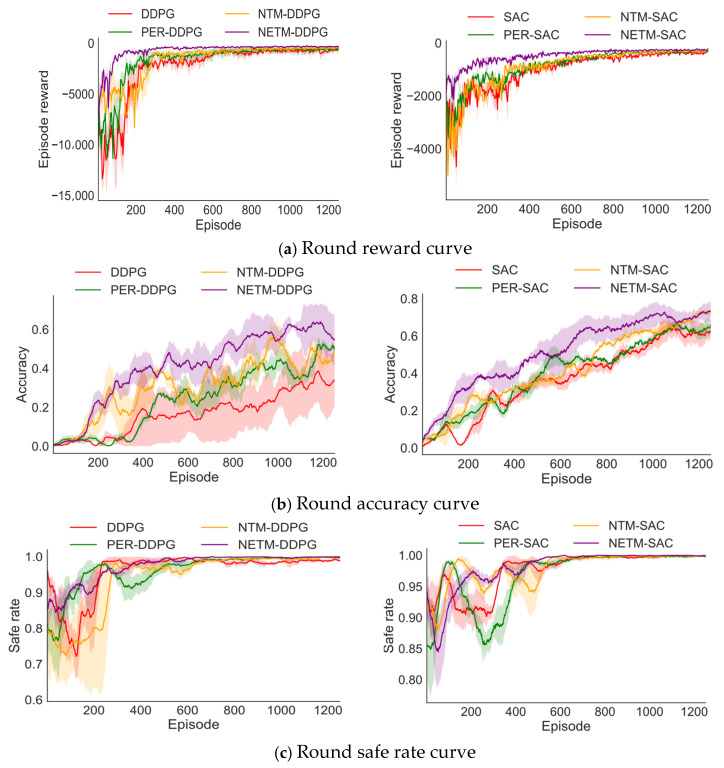
Performance comparison of different algorithms in Scenario 1.

**Figure 7:**
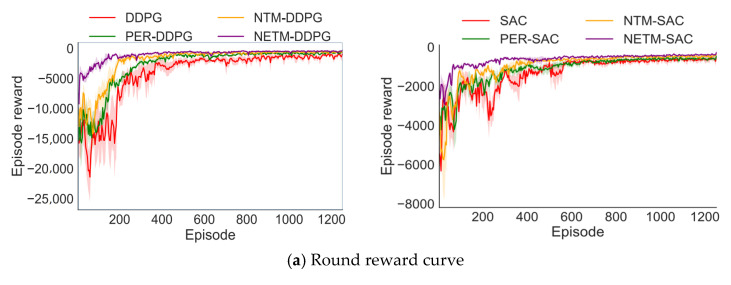

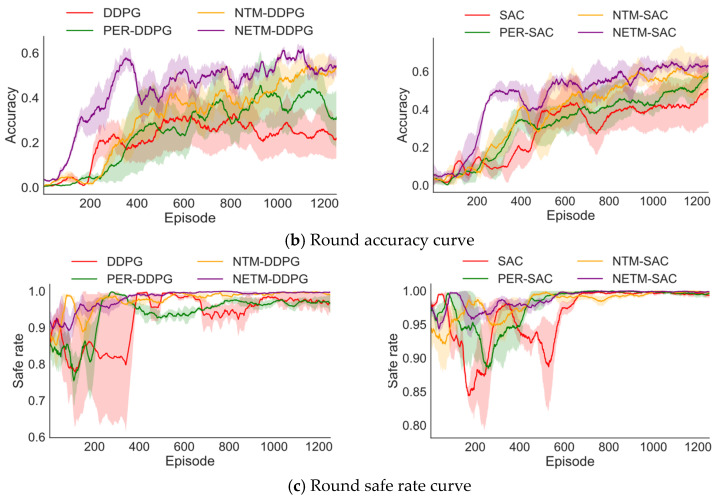
Performance comparison of different algorithms in Scenario 2.

First, this section conducts comparative experiments on four methods: original DRL, PER-DRL, NETM-DRL, and NTM-DRL in different scenarios based on two representative DRL algorithms (DDPG and SAC) using the original reward function OSR. For NETM-DRL, before training starts, the GAIL algorithm is first used to augment 10,000 expert experiences to initialize the self-increasing expert experience replay pool. NTM-DRL is the NETM-DRL method with the self-increasing expert experience replay pool removed. In addition, in PER-DRL, the selection of the priority sampling index $\alpha_{per}$ has a certain impact on the algorithm’s performance. Therefore, this paper selects different $\alpha_{per}$ for the experiments and then only displays PER-DRL with the optimal performance. For each method, this paper reports the average performance of the algorithm under three random seeds. Figure 6 and Figure 7 show the performance comparison diagrams of different algorithms during training in Scenario 1 and Scenario 2, and Table 2 shows the evaluation index results after the algorithm trains for 1000 episodes, where $R_{c}$, $A_{c}$, and $S_{c}$ are the average episode reward, average accuracy, and average safe rate after 1000 episodes of algorithm training, respectively.

As shown in Figure 6 and Figure 7 and Table 2, for both scenarios, all DRL algorithms based on NETM show faster convergence speed, higher accuracy, and a safer rate than the benchmark algorithms. From the episode reward, it can be seen that in the early training stage, the episode reward values of NETM-DDPG and NETM-SAC quickly reached a high level. This indicates that the quality of the augmented experience data in the self-increasing expert experience replay pool has reached the expert level, which can provide good expert strategy guidance for the robotic arm, enabling the robotic arm to learn dynamic obstacle avoidance strategies in a very short time.

**Figure 8:**
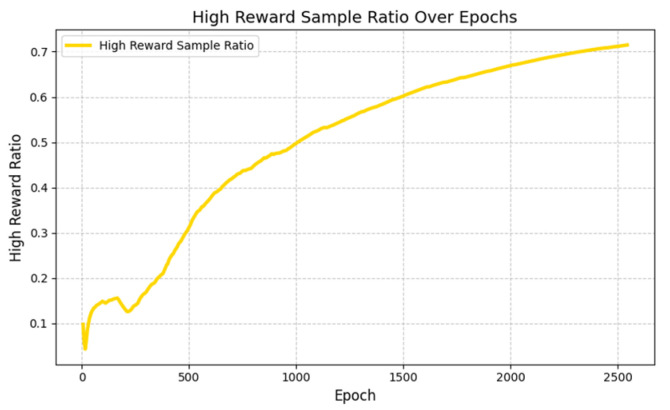
Variation trend of sampling ratio of high-reward samples during training episodes.

Figure 8 shows the variation trend of the sampling ratio of high-reward samples (including high-quality experiences in the self-increasing expert experience pool and valid experiences in the successful experience pool) under the NETM mechanism with training episodes in Scenario 2 (dynamic target–dynamic obstacle environment). It can be seen from the figure that the sampling ratio of high-reward samples shows a gradual upward trend with the training process: in the early stage of training (0–500 epochs), the ratio remains between 0.2 and 0.3. At this time, the neural network is still learning the basic sampling strategy, and it is necessary to balance the proportion of expert experiences, successful experiences, and failed experiences to avoid overfitting; as the training progresses (500–2000 epochs), the ratio gradually increases to 0.5–0.6, indicating that the neural network has autonomously learned the rule that “high-reward samples are more valuable” through the algorithm’s reward value and the loss value of the policy network (as described in Section 3.1.2), and starts to prioritize sampling experiences that are more effective for policy optimization; in the later stage of training (after 2000 epochs), the ratio stabilizes at around 0.6. At this time, the algorithm is close to convergence, and the continuous input of high-value experiences further consolidates the stability of the policy.

This trend is highly consistent with the performance of NETM-SAC and NETM-DDPG in Figure 7: the dynamic increase in the proportion of high-reward samples enables the algorithm to quickly obtain expert strategy guidance in the early stage, accelerate convergence through high-quality experiences in the middle stage, and maintain high performance through the stable input of high-value experiences in the later stage. Eventually, it achieves a higher accuracy and safety rate than PER-DRL and the original DRL. This result intuitively verifies the effectiveness of the “neural network-based dynamic sampling strategy” in NETM—without manually setting the sampling ratio or introducing additional hyperparameters, the maximum utilization of experience can be achieved only by learning the reward and loss signals of the algorithm itself, providing a mechanism-level explanation for the performance advantages of NETM in Figure 7.

In the later training stage, the cumulative reward value obtained by NETM-DRL is the highest. In addition to the guidance of expert strategies, it is also because there is a Chain Effect in DRL training, that is, the improvement of the algorithm’s early performance will chain-effect the training performance of the algorithm in the middle and later stages, enabling the algorithm to obtain higher cumulative reward values. Finally, the accuracy and safe rate in both environments further verify the effectiveness of expert experience guidance.

Secondly, this paper discusses the impact of the neural network-based experience sampling strategy on the DRL algorithm. It can be seen that, except for NETM-DRL, the improvement effect of NTM-DDPG and NTM-SAC is the highest, indicating that dynamically learning successful and failed experiences at different ratios in different training stages can improve the utilization rate of data experience, which is very helpful for the robotic arm’s training. Although in Scenario 1, the early convergence speed of NTM-DRL is slower than that of PER-DRL and original DRL because the neural network needs a certain time to learn good sampling ratios, the final performance of NTM-DRL is optimal.

Finally, the improvement effect of PER-DDPG is the worst, especially in Scenario 1, indicating that in simple scenarios or with dense reward functions, the PER acceleration effect is not significant. In addition, the balance factors $\alpha_{per}$ corresponding to PER in the two environments are different, and the value of $\alpha_{per}$ needs to be continuously adjusted in the experiment, which increases the difficulty of parameter tuning.

#### 4.3.2. Comparison of Time Complexity of Different Experience Replay Mechanisms

Next, this paper compares the time complexity of six algorithms, including NETM-DDPG, PER-DDPG, DDPG, NETM-SAC, PER-SAC, and SAC, from three aspects: average sampling time, average update time, and average total time per time step.

It can be seen from Table 3 that PER-DDPG has the highest time complexity because in the sampling stage, PER-DDPG selects experiences according to priority and simultaneously calculates the importance sampling weights of experiences to reduce the bias introduced by priority sampling. In the update stage, PER-DDPG also needs to update the priority of experience data in the experience pool according to the TD error. Compared with PER-DDPG, the NETM-DDPG algorithm has lower time complexity, and as proved by the above comparative experiments, it can achieve good results. Meanwhile, due to the complexity of the SAC algorithm’s own structure and its relatively large computational overhead, the overall time is higher than that of the DDPG algorithm. Unlike DDPG, the introduction of the NETM and PER mechanisms does not consume more time. This is because the goals of SAC and DDPG are different. In pursuit of the goal of high sample efficiency, both PER and NETM play a certain promoting role.

#### 4.3.3. Comparison of Reward Function Training Experimental Effects

**Figure 9:**
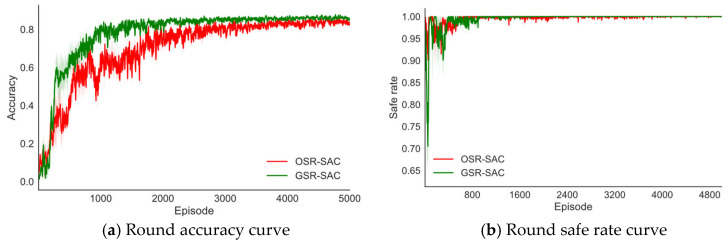
Performance comparison of different algorithms in difficult scenario 2.

Finally, to verify the feasibility of the proposed GSR, this paper conducts a comparative experiment on the difficult Scenario 2 using the original reward function OSR and the GSR based on the SAC algorithm using the NETM experience replay mechanism. For each method, this section also reports the average performance of the algorithm under three random seeds. Figure 9 shows the performance comparison results of different algorithms during training.

As shown in Figure 9, the performance of the GSR-SAC algorithm is significantly improved compared with OSR-SAC. In terms of accuracy, the convergence speed of the GSR-SAC algorithm is faster and the fluctuation amplitude is smaller than that of OSR-SAC, and the convergence can obtain higher accuracy. This is because the target position is always moving, and when the robotic arm uses the original reward function, the speed of the end-effector and the target position is not considered, even if it enters the area near the target position at a certain time step, it may be thrown away by the moving target at the next time step due to the different speeds, which will reduce the accuracy of the task and increase the fluctuation of accuracy. By adding the velocity information of the end-effector and the target position to the reward function, the GSR makes the movement trend of the manipulator consistent with the target position while tracking to the target position, which improves the performance of the trajectory planning algorithm.

In addition, when the robotic arm is near the target position, the change in $d_{T}$ will be very small, which will cause the robotic arm to be insufficiently sensitive to the subtle position changes of the end-effector, also exacerbating this situation. The additional reward in GSR increases the robotic arm’s sensitivity to the tiny changes in $d_{T}$, further improving the robotic arm’s performance.

Finally, although the safe rate of GSR-SAC is lower than that of the OSR-SAC algorithm in the early training stage, after 2000 episodes of training, the average number of episodes with collisions for GSR-SAC under different random seeds is 3, while that for OSR-SAC is 170. It can be seen that canceling the range of the repulsive potential field of dynamic obstacles can endow the robotic arm with the ability to globally perceive obstacles, thereby enabling the robotic arm to learn effective dynamic obstacle avoidance strategies.

**Figure 10:**
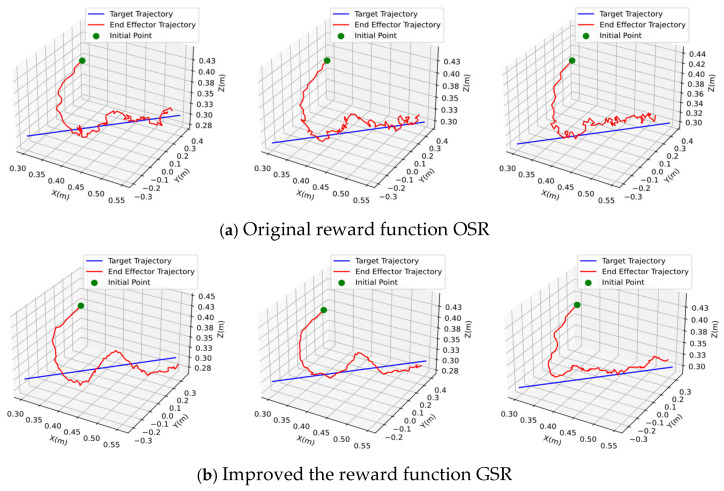
Comparison of trajectories generated by different algorithms.

In addition, in order to prove the actual effect of GSR, this paper uses different reward mechanisms to simulate multiple times under the same conditions, based on the SAC algorithm, and then shows the paths planned by the two methods when the accuracy rate is 83% and the safe rate is 100%. As shown in Figure 10, three representative sets of simulation results were selected for each method.

In the figure, the green origin represents the initial position of the end-effector of the robotic arm, the red line is the trajectory planned by the algorithm for the end-effector, and the blue line is the trajectory of the target. It can be seen that under the same accuracy and safe rate, the path planned by the DRL algorithm based on the GSR reward function is smoother and more natural.

## 5. Conclusions

This paper discusses the problem of dynamic obstacle avoidance trajectory planning for robotic arms based on DRL. First, a NETM is proposed. This mechanism divides the original experience pool into a successful experience replay pool and a failed experience replay pool, then uses GAIL to expand a small amount of human demonstration experience and successful experiences generated by the algorithm into a large number of expert experiences, which are stored in the self-increasing expert experience pool. During training, a neural network-based experience sampling mechanism is used to extract data from the three experience pools at different ratios in different stages for learning. This method provides high-quality data throughout training, addressing the challenge of acquiring expert experiences, and significantly enhances experience utilization. Experimental results show that the DRL algorithm loaded with NETM has faster convergence speed and higher convergence performance.

In addition, this paper adds velocity constraints between the end-effector and the moving target to the gravitational potential field of the artificial potential field method and cancels the repulsive force influence range of dynamic obstacles. Then, an improved GSR is proposed based on this. Experimental results show that GSR can bring faster convergence speed, higher success rate, and a safer rate to the algorithm. In addition, under the same conditions, the trajectory generated by the algorithm based on GSR is smoother and more natural.

Although the research in this paper has achieved significant results in trajectory planning in dynamic environments, it still faces some challenges to be overcome. The existing experiments are carried out based on the controllable environment of the Pybullet simulation platform, where the kinematic model, sensor noise, and physical interactions of the robotic arm are all simplified, which differ from complex factors such as mechanical vibrations and delayed responses in real industrial scenarios. Secondly, the efficiency of the algorithm demonstrated in the simulation environment (such as the fast convergence of NETM and the low collision rate of GSR) needs to be further verified on real hardware. In actual deployment, problems such as limited computing resources and interference from sensor data noise may be encountered, leading to a decline in the accuracy and real-time performance of trajectory planning. Future research should focus on promoting the migration from simulation to real hardware and improving the deployment capability in practical applications by optimizing the lightweight level of the algorithm and enhancing environmental robustness.

## Figures and Tables

**Table 1:** Lists the main hyperparameters used in the experiments. In addition, this paper runs the code and completes all experiments on a Tesla A100 GPU.

Parameter	Value	Parameter	Value
Actor network learning rate	0.00015	Memory pool capacity	1,000,000
Critic network learning rate	0.0015	ε	0.05
Reward discount factor	0.99	$c_{1}$	250
Soft update rate	0.001	$c_{2}$	15
Batch_size	64	n	35
Expert memory pool capacity	10,000	τ	0.2
Maximum time steps	100	δ	4

**Table 2:** Evaluation index results of different algorithm training processes.

Model	Environment 1			Environment 2		
	$R_{c}$	$A_{c}$	$S_{c}$	$R_{c}$	$A_{c}$	$S_{c}$
NTM-DDPG	−804	44.68%	99.80%	−856	50.76%	99.57%
NETM-DDPG	−405	59.93%	99.43%	−616	55.89%	99.68%
DDPG	−1161	32.15%	98.81%	−2144	22.18%	97.40%
PER-DDPG	−885	43.22%	99.81%	−1209	34.30%	96.93%
NTM-SAC	−565	67.25%	99.85%	−636	57.94%	99.86%
NETM-SAC	−360	70.51%	99.96%	−500	62.69%	99.86%
SAC	−701	61.43%	99.88%	−939	43.35%	99.58%
PER-SAC	−596	63.26%	99.82%	−808	50.26%	99.65%

**Table 3:** Comparison of time complexity of different algorithms.

Method	Total Time (ms)	Sampling Time (ms)	Update Time (ms)
NETM-DDPG	1356.21	6.17	1007.65
DDPG	1306.08	4.84	974.82
PER-DDPG	1456.39	31.51	1269.87
NETM-SAC	1986.57	137.35	1754.36
SAC	2246.11	148.21	2002.02
PER-SAC	2037.74	141.53	1797.97

## Data Availability

The original contributions presented in this study are included in the article. Further inquiries can be directed to the corresponding author.
